# Supplementary material for: Identification of epigenome-wide DNA methylation differences between carriers of APOE ε4 and APOE ε2 alleles
Source: Genome Med. 2021 Jan 4;13:1. doi: 10.1186/s13073-020-00808-4 (PMC7784364; doi:10.1186/s13073-020-00808-4)
Supplement: Supplementary file 2 — Additional file 2: Fig. S1. Scree plot showing the eigenvalues of the first 50 genetic principal components for the Generation Scotland: Scottish Family Health Study. Fig. S2. The genomic region encompassing APOE, which contains 16 of the APOE ε4 vs. ε2-associated DMPs identified by meta-analysis (chr19: 45,221,584 – 45,454,752; GRCh37/hg19). Fig. S3. Bar charts showing the mean methylation levels (M-values) in the discovery sample for the 20 meta-DMPs split by APOE genotype (ε2/ε2, ε2/ε3, ε3/ε3, ε3/ε4, and ε4/ε4). Fig. S4. The genomic regions containing the six identified DMRs. [file 13073_2020_808_MOESM2_ESM.pdf]

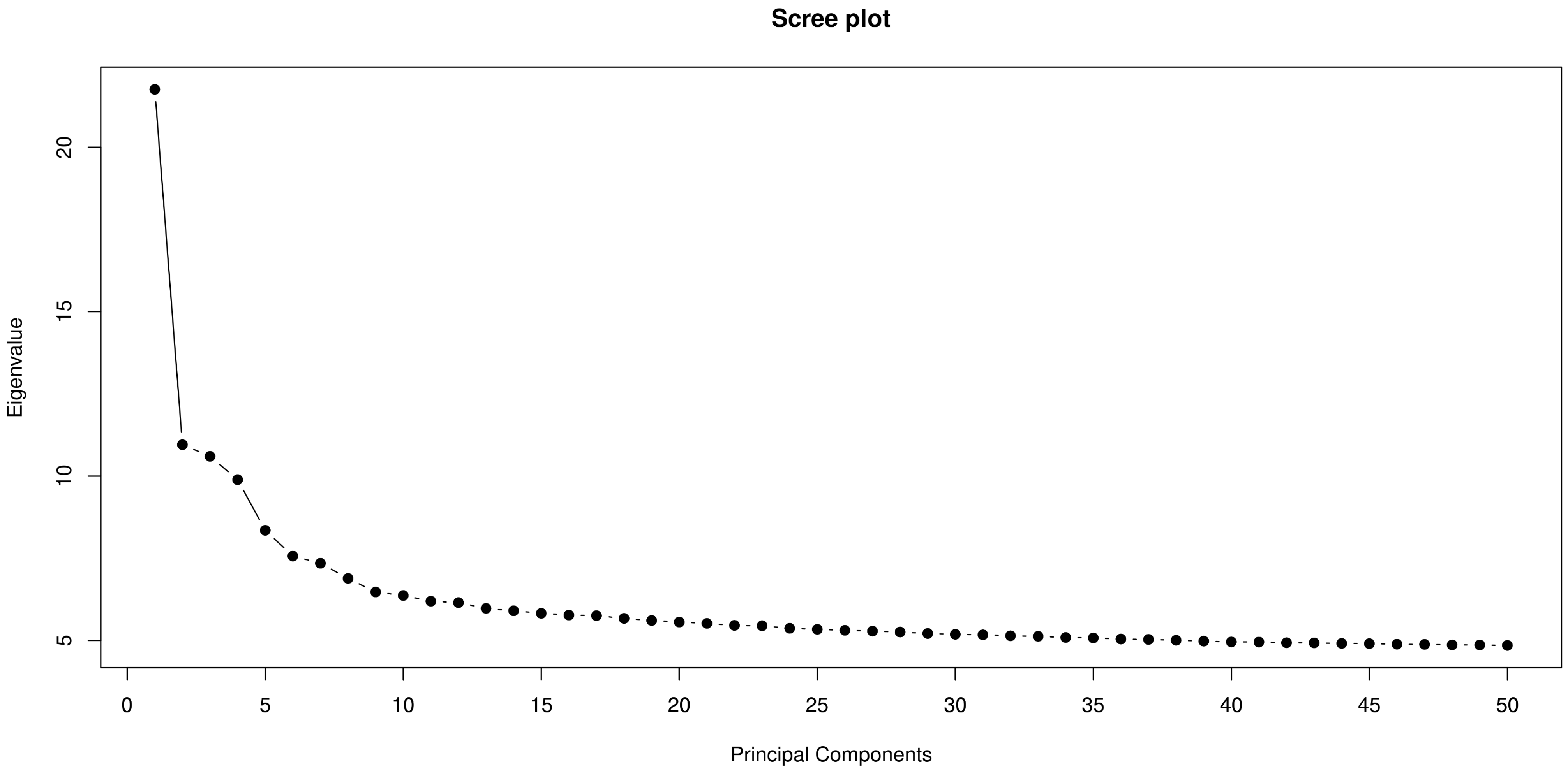

Fig. S1. Scree plot showing the eigenvalues of the first 50 genetic principal components for the Generation Scotland: Scottish Family Health Study.

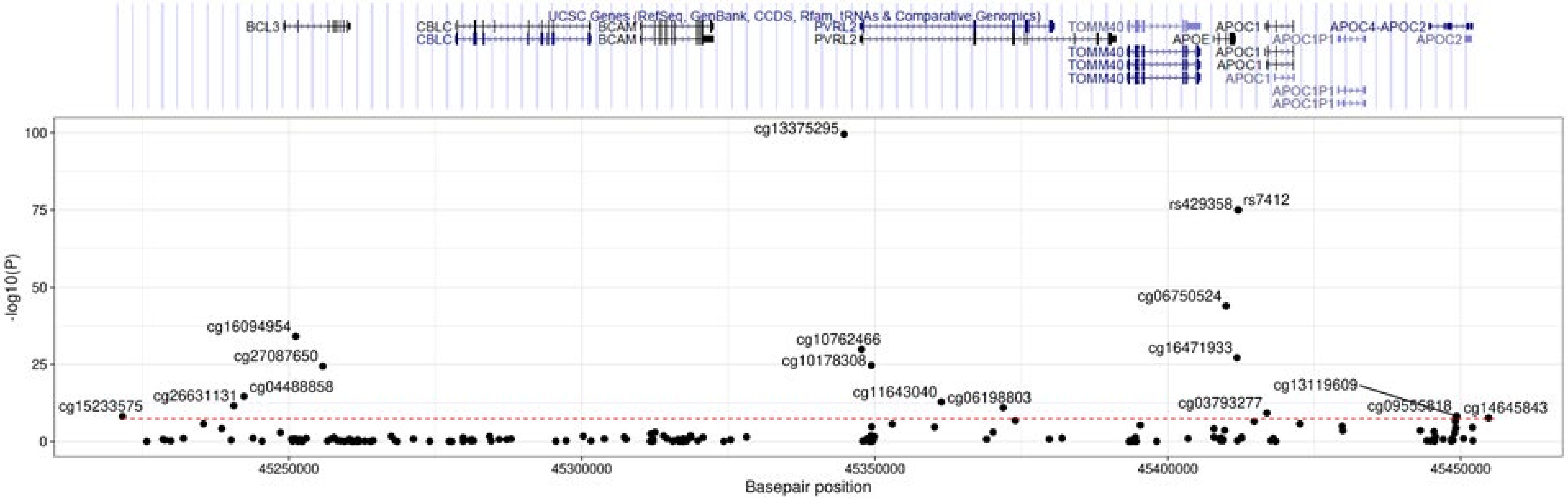

Fig. S2. The genomic region encompassing *APOE*, which contains 16 of the *APOE* ε4 vs. ε2-associated DMPs identified by meta-analysis (chr19: 45,221,584 – 45,454,752; GRCh37/hg19). The upper panel, downloaded from the UCSC Genome Browser (GRCh37/hg19) indicates the location of genes (NCBI RefSeq gene track). The lower panel indicates CpG sites in the region. The position of the CpG on the y-axis is determined by the $–\log_{10}$ of its meta-analysis *P*-value and its position on the x-axis is determined by its genomic location. The dashed red line indicates the threshold for significance ($p \leq 3.6 \times 10^{-8}$) and CpGs attaining significance are labelled with their identifier. The two *APOE* SNPs, rs429358 and rs7412, which define the *APOE* ε2/ε 3/ε4 haplotype are also indicated.

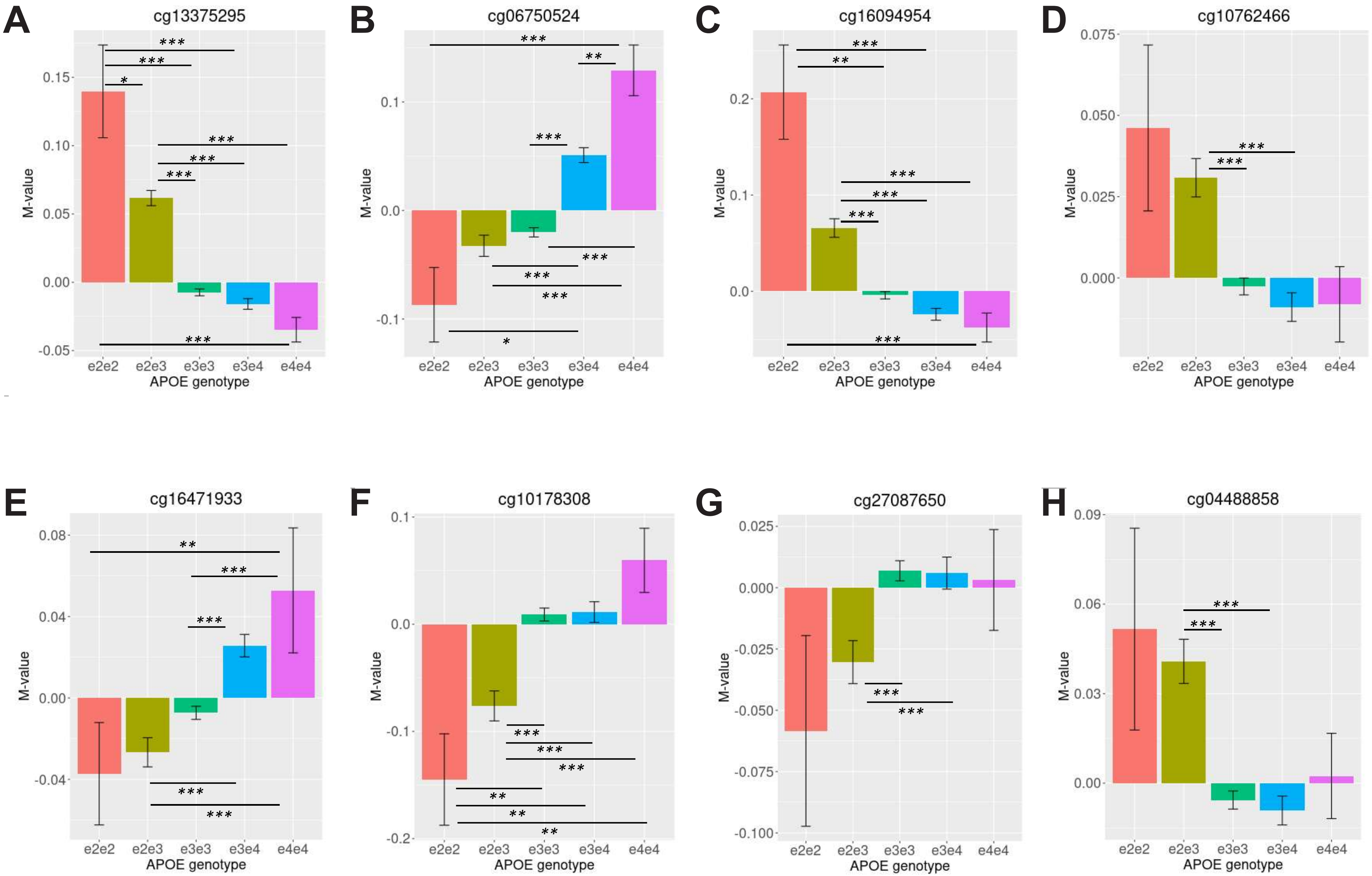
A
cg13375295
B
cg06750524
C
cg16094954
D
cg10762466
E
cg16471933
F
cg10178308
G
cg27087650
H
cg04488858
M-value
APOE genotype
e2e2
e2e3
e3e3
e3e4
e4e4

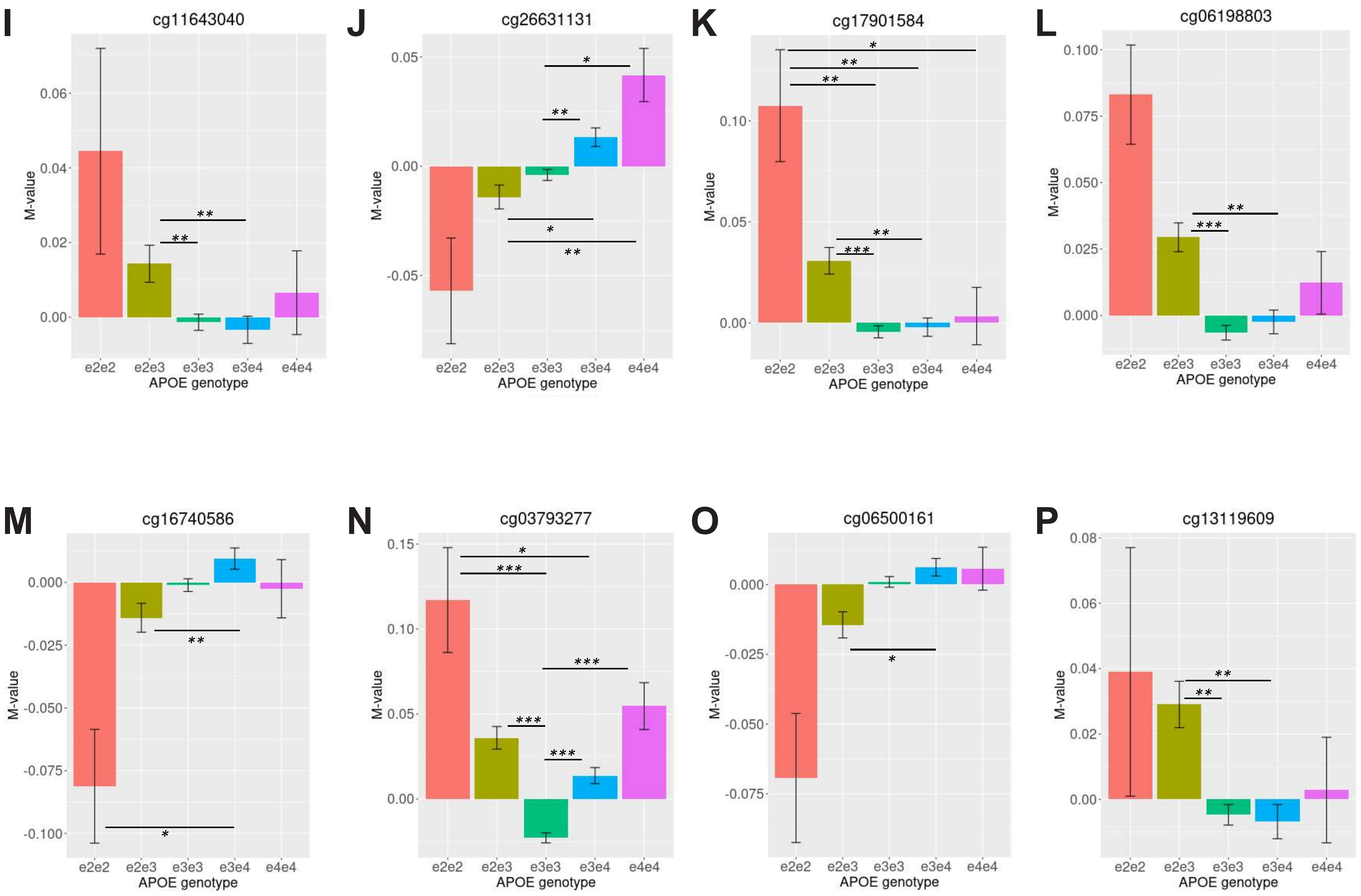
I
cg11643040
M-value
APOE genotype
e2e2
e2e3
e3e3
e3e4
e4e4
J
cg26631131
K
cg17901584
L
cg06198803
M
cg16740586
N
cg03793277
O
cg06500161
P
cg13119609

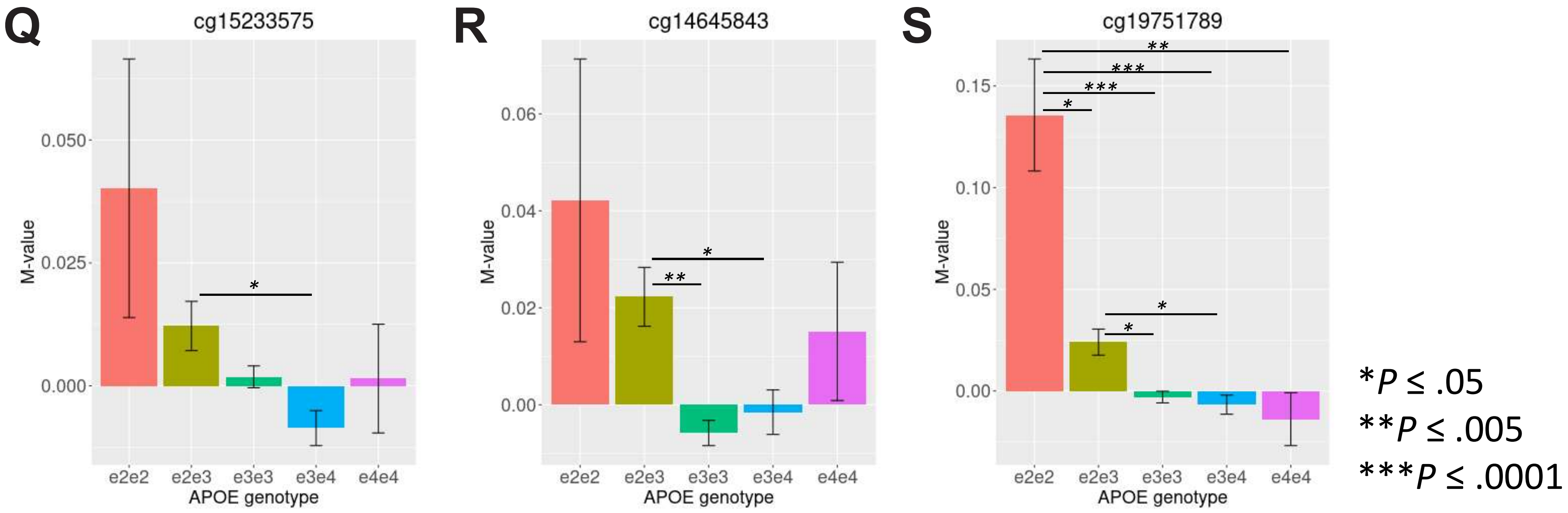

Fig. S3. Bar charts showing the mean methylation levels (M-values) in the discovery sample for the 20 meta-DMPs split by *APOE* genotype (ε2/ε2, ε2/ε3, ε3/ε3, ε3/ε4, and ε4/ε4). Error bars indicated standard errors of the mean. Pairwise differences were assessed using Tukey's test of Honestly Significant Differences and significant differences are indicates by asterisks (**P* ≤ .05, ***P* ≤ .005, ****P* ≤ .0001).

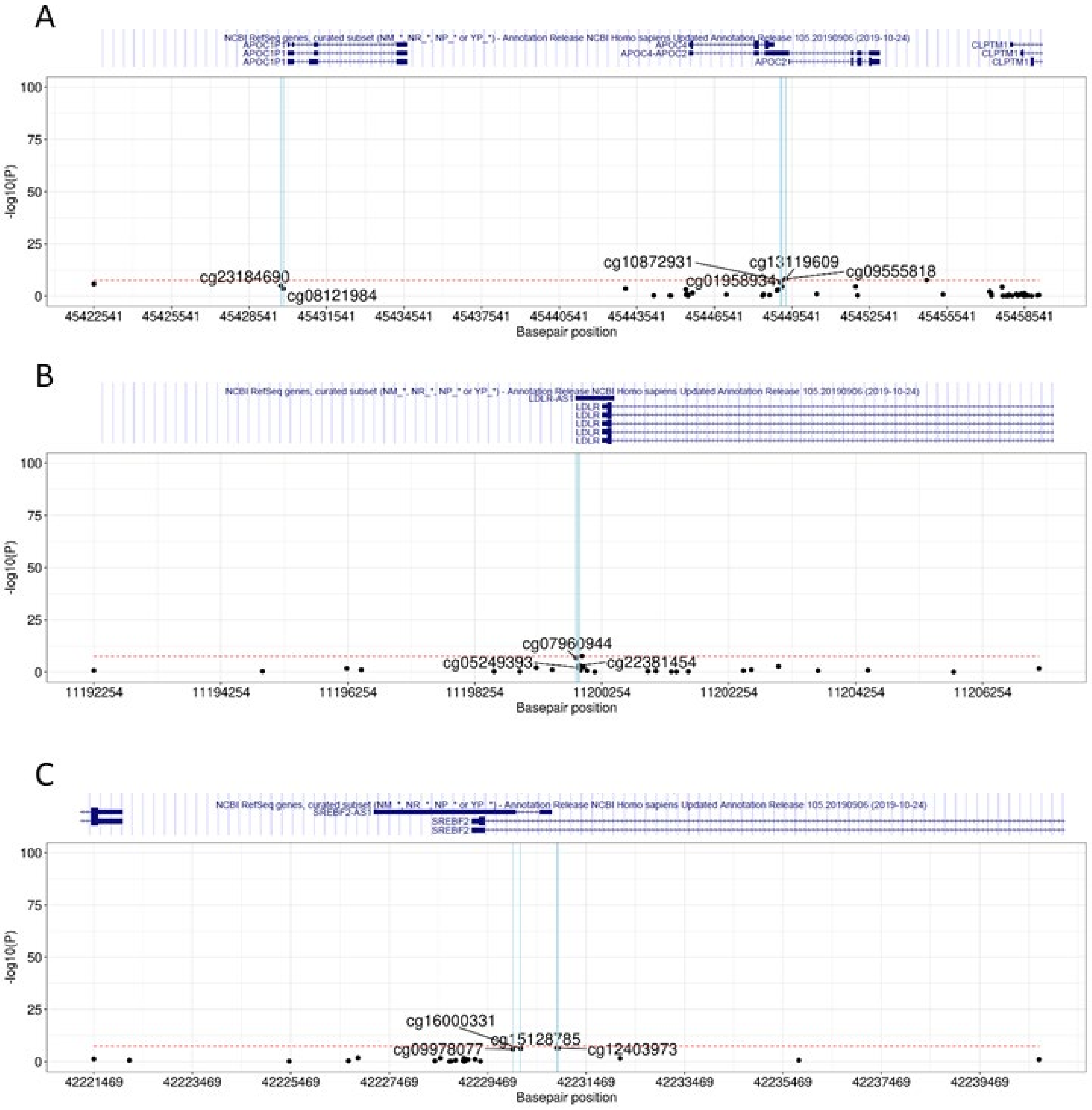

Fig. S4. The genomic regions containing the six identified DMRs. **A**. indicates the three DMRs within the *APOE* locus (i. cg01958934 and cg10872931; ii. cg01958934 and cg10872931; iii. cg23184690 and cg08121984); **B**. shows the *LDLR* DMR; and **C**. shows the two *SREBF2* DMRs (i. cg15128785 and cg1240397; ii. cg09978077 and cg16000331). Each plot shows an extended region spanning from 10 kb downstream of the most 5’ DMR CpG to 10 kb upstream of the most 3’ DMR CpG. In each plot, the upper panel, downloaded from the UCSC Genome Browser (GRCh37/hg19) indicates the location of genes (NCBI RefSeq gene track). The lower panel indicates CpG sites in the region. The position of the CpG on the y-axis is determined by the $-\log_{10}$ of its meta-analysis *P*-value and its position on the x-axis is determined by its genomic location. The dashed red line indicates the threshold for significance ($p \leq 3.6 \times 10^{-8}$) in the EWAS. The vertical blue lines indicate the locations of CpGs that are involved in a DMR and these CpGs are labelled with their probe ID.
